# The N-shaped orthotopic ileal neobladder: functional outcomes and complication rates in 119 patients

**DOI:** 10.1186/s40064-016-2287-1

**Published:** 2016-05-17

**Authors:** Thomas De Sutter, Murat Akand, Maarten Albersen, Wouter Everaerts, Ben Van Cleynenbreugel, Dirk De Ridder, Hans Goethuys, Lisa Moris, Uros Milenkovic, Hendrik Van Poppel, Frank Van Der Aa, Steven Joniau

**Affiliations:** Department of Urology, University Hospitals Leuven, Herestraat 49, 3000 Leuven, Belgium; Department of Urology, School of Medicine, Selcuk University, Konya, Turkey; Department of Urology, Ziekenhuis Oost-Limburg, Genk, Belgium

**Keywords:** Cystectomy, Ileal orthotopic neobladder, Bladder substitution, Complications, Continence

## Abstract

**Background:**

We report our long-term experience with 119 cases of N-shaped orthotopic ileal neobladder.

**Methods:**

Between March 1996 and July 2013, a total of 119 patients (102 men, 17 women) underwent cystectomy with creation of an N-shaped orthotopic ileal neobladder. The Clavien–Dindo classification score was used for grading early (<3 months postoperative), late, and pouch-related and non-pouch-related complications. Daytime and nighttime continence were evaluated for male and female patients separately, with patients subdivided in three groups: completely continent, use of ≤1 pad, and use of >1 pad.

**Results:**

Median follow-up was 75 months (range 3–204). Early complications (15 major, 54 minor) occurred in 39.5 % of 119 patients whereas 53.1 % presented with late complications (56 major, 39 minor; 111 patients evaluated). Urinary infection and outlet obstruction were both the most frequent early and late pouch-related complications; early non-pouch-related complications were mainly infectious and gastrointestinal, and the most common late non-pouch-related problem was wound herniation. At 12 months, 96 and 60 % of the men and 84.6 and 66.7 % of the women respectively achieved daytime and nighttime continence.

**Conclusion:**

Complication rates of the N-shaped orthotopic ileal neobladder were relatively high, probably because of meticulous recording and follow-up. Daytime continence rates were better than nighttime rates. N-shaped orthotopic ileal neobladder can be a good option for urinary diversion in selected patients who undergo radical cystectomy.

## Background

Radical cystectomy (RC) with pelvic lymph node dissection and urinary diversion is the standard therapy for non-metastatic muscle-invasive bladder cancer and high-risk non-muscle-invasive bladder cancer (Witjes et al. 2014). Over the last two decades, orthotopic ileal neobladder (OIN) has gained popularity, with the Hautmann and Studer pouches used commonly (Hautmann 2003; Hautmann et al. 2013). Compared to the ileal conduit (IC), it offers similar cancer control rates and possibly a higher quality of life by preserving continence and near-normal voiding function, avoiding urinary stoma (Yossepowitch et al. 2003; Hautmann et al. 2006; Studer et al. 2006). Disadvantages are the more complex surgical procedure, higher risk of postoperative complications, and a significant risk of (nighttime) incontinence or even hypercontinence, the latter especially in women (Hautmann et al. 1999; Arai et al. 1999). The importance of intensive pelvic floor re-education to reduce incontinence rates has been acknowledged (Arai et al. 1999). Orthotopic bladder substitution (OBS) is therefore indicated only for highly motivated and cognitively capable patients. In neobladder creation, different parts of the gastrointestinal tract have been variously suggested for use (Hinman 1998). At present, a detubularized segment of ileum is recommended (Hautmann 2003; Hautmann et al. 2013), offering a low-pressure reservoir with good capacity (Hautmann et al. 2013; Nam et al. 2013; Singh et al. 2014).

In 2005, we published our early results in 58 patients undergoing RC and OBS with the N-shaped ileal neobladder, with a mean follow-up of 38 months (Joniau et al. 2005). We now report a series of 119 consecutive patients and describe long-term experience with the Leuven N-pouch technique, creating an ileal pouch with an isoperistaltic afferent limb onto which both ureters are anastomosed in an end-to-end fashion. This technique combines features of the popular Hautmann and Studer neobladders, creating a good-capacity pouch with an active anti-reflux mechanism (Hautmann et al. 1988; Studer et al. 1989). The aim of this retrospective study was primarily to assess our long-term experience in terms of complication rates and continence.

## Methods

### Patient population and study design

Between March 1996 and July 2013, a total of 810 RCs were performed at our tertiary referral institution (UZ Leuven, Belgium), and 119 consecutive patients (102 men and 17 women) underwent cystectomy with N-shaped OIN reconstruction. We reviewed their medical records retrospectively in accordance with the ethical standards laid down in the 1964 Declaration of Helsinki and its later amendments. The confidentiality of patient data was guaranteed.

Absolute contraindications were prostatic stromal tumor invasion, inflammatory bowel disease, and impaired renal function (serum creatinine >2.0 mg/dL) and liver function. Other (relative) exclusion criteria were poor physical condition, salvage cystectomy, extensive local tumor burden, cognitive incapability, and lack of motivation or physical inability to perform intermittent catheterization.

### Follow-up

Database collection was based on outpatient and admission reports until June 2015. Standard outpatient visits took place at 3-month intervals during the first two postoperative years, at 6-month intervals up to the fifth year, and yearly thereafter. Physical examination was performed at every visit, as were laboratory tests (blood count, renal and liver function, sodium, potassium, chloride, bicarbonate, and vitamin B12 from the third year on) and imaging. Abdominal ultrasound and chest X-ray were alternated with abdominal and thoracic computed tomography scans.

### Surgical technique

Cystoprostatectomy was performed in men while women underwent cystectomy with sparing of the urethropelvic ligament and the neurovascular structures of the urethra. Of the 17 women, 9 had anterior exenteration while 8 had cystectomy alone. A bilateral pelvic lymph node dissection was performed for any patient with an indication of oncological disease.

**Fig. 1:**
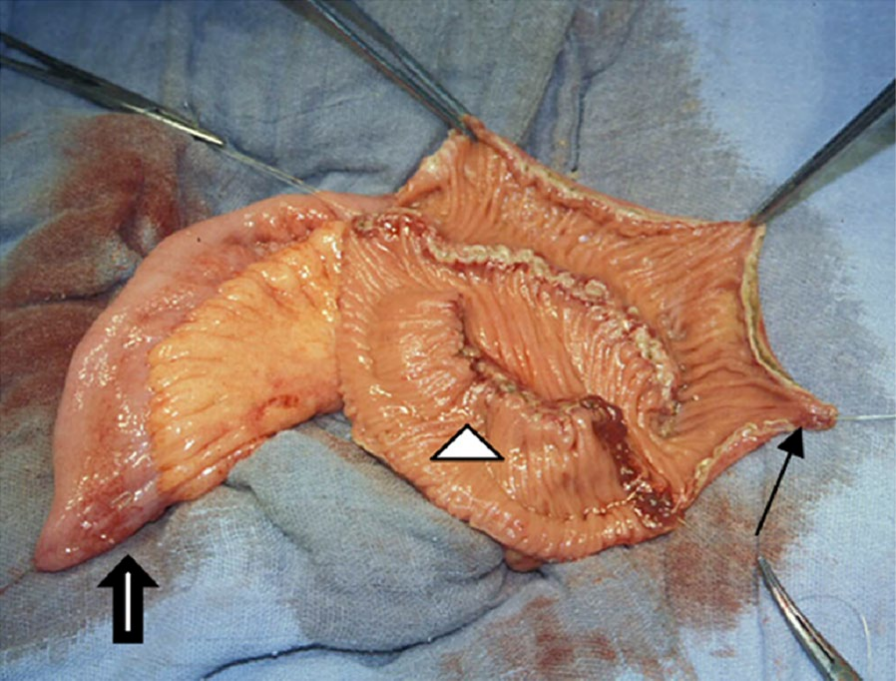
The N-shaped orthotopic ileal neobladder. *Large arrow* Intact afferent isoperistaltic segment, acting as an active anti-reflux mechanism. The ureters are anastomosed to this segment in an end-to-end manner. *Arrowhead* Three antimesenterically opened ileal segments, creating the N-shaped pouch. *Small arrow* The lowest point of the pouch, forming the urethral anastomosis

Subsequently, an N-shaped neobladder was created with a 50-cm segment of preterminal ileum. This segment was folded into four equally long parts, three of them forming the N-shaped pouch while the most proximal part remained intact, forming the afferent isoperistaltic limb onto which the ureters were anastomosed in an end-to-end fashion separately (Fig. 1). An exact description of this technique has been published previously (Joniau et al. 2005).

### Complications

Complications were registered as early (occurring within 3 months postoperative) or late, as well as pouch-related or non-pouch-related. The validated Clavien–Dindo classification score (CCS) was used to grade complications (Dindo et al. 2004). CCS I and II were considered to indicate minor complications and CCS III to V major complications. In addition, the treatment for these complications was noted, in particular conservative, endoscopic, or open surgical therapy.

### Continence

Continence was assessed during follow-up visits. For subdividing the patients according to their continence status, we used the UCLA-Prostate Cancer Index with a minor modification (Litwin et al. 1998). In this way, continence was defined with more strict criteria, and patients were divided into three groups: Group 1 (G1), patients without any need for protection; Group 2 (G2), those in need of maximally one (safety) pad; and Group 3 (G3), those using more than one pad, a diaper, or a condom catheter. Daytime continence (DTC) and nighttime continence (NTC) were evaluated separately.

### Voiding pattern and catheterization

Micturition was evaluated primarily at consultation visits, where information about urinary loss, catheterization need and spontaneous voiding was acquired. Clean intermittent self-catheterization was started for those with a post-micturitional residue of more than 150 mL or recurrent urinary tract infections (UTIs).

### Statistical analysis

Descriptive analyses were performed with SPSS version 15.0 (SPSS Inc., Chicago, Illinois, USA), and were given as median and range for continuous quantitative variables and as percentages for non-numeric variables.

## Results

### Population

The median age of the men was 59 years (range 34–74); for women, it was 55 years (range 29–68). Median follow-up was 75 months (range 3–204 months).

Elective OBS was performed in 118 patients; one patient undergoing bilateral distal ureterectomy suffered a rupture of a very fibrotic bladder wall, and subsequent cystectomy with ileal neobladder reconstruction was performed. Bladder transitional cell carcinoma was the operative indication in the vast majority of patients (95.7 %). Only 30 patients (25.2 %) (25 men and 5 women) had a nerve-sparing surgery (23 bilateral, 7 unilateral).

Five patients presented preoperatively with positive lymph nodes, four of whom were treated with neo-adjuvant chemotherapy. Another 14 patients with muscle-invasive bladder cancer received neo-adjuvant chemotherapy. Patient demographics and disease characteristics are described in Table 1. Because of cancer-related and other cause deaths, the short- and long-term follow-up data differ.

**Table 1:** Patient demographics, disease characteristics, procedural data, and pre- and postoperative pathology results

Number of patients (Male/Female)	119 (102/17)
Age (years)	Male: 59 (34–74) Female: 55 (29–68)
Follow up (months)	75 (3–204)
Operative indication	
Transitional cell carcinoma	113 pts
Interstitial cystitis	2 pts
Vesicorectal fistula	1 pt
Prostate cystadenoma	1 pt
Persistant leakage from continent vesicostomy	1 pt
Bladder wall calcification after BCG	1 pt
Operation time (min)	210 (120–360)
Blood loss (mL)	1100 (100–3600)

T stage	Preoperative	Postoperative	Positive LN
T0	3 (2.7 %)	29 (25.7 %)	1/29 (3.4 %)
Tx	3 (2.7 %)	0 (0 %)	0 (0 %)
Tis	5 (4.4 %)	9 (7.9 %)	0/9 (0 %)
Ta	1 (0.8 %)	5 (4.4 %)	0/5 (0 %)
T1	22 (19.5 %)	15 (13.3 %)	1/15 (6.7 %)
T2	75 (66.4 %)	22 (19.5 %)	4/22 (18.2 %)
T3	4 (3.5 %)	29 (25.7 %)	12/29 (41.4 %)
T4	0 (0 %)	4 (3.5 %)	3/4 (75 %)
Total	113 (100 %)	113 (100 %)	21/113 (18.6 %)

Data are given as either median (range) or *n* (%)

### Complication rates

Of the 119 patients, 47 (39.5 %) had early complications (≤3 months), and 59 of 111 evaluable patients (53.1 %) had late complications (>3 months). One patient died in the perioperative period due to perforation of the small intestine, which resulted in septic shock and acute myocardial infarction. Seven other patients had no long-term follow-up (>3 months) because of death within the first year (>3 months) (5 patients; 4 cancer-related and 1 death at home for unknown reasons), follow-up abroad (1 patient), or loss to follow-up (1 patient). All complications were counted separately, and some patients experienced more than one complication.

#### Early complications

Table 2 gives an overview of the early complications and their management. In 119 patients, we registered 15 major early complications (CCS III–V) compared to 54 minor early complications (CCS I–II). Early pouch-related complications occurred in 23 patients (19.3 %). UTI was the most frequent, occurring in 17 patients (14.3 %), with 14 cases classified as minor and 3 that led to urosepsis with intensive care admission. Non-pouch-related early complications occurred in 33 patients (27.7 %). Minor complications were diverse and almost equally distributed among gastrointestinal (8.4 %), pulmonary (6.7 %), and wound-related problems (5 %). Six patients (5 %) presented with major complications, and open surgical exploration was necessary in four of them because of small bowel perforation, mesenterial bleeding, evisceration and removal of a textiloma.

**Table 2:** Overview of early complications (≤3 months) (pouch and non-pouch-related) by the Clavien–Dindo classification in 119 patients

Clavien–Dindo score (CCS)	Number of complications (%)
Major	
V	1 (0.8 %)
IV	
a	0 (0 %)
b	3 (2.5 %)
III	
a	3 (2.5 %)
b	8 (6.7 %)
Minor	
II	34 (28.6 %)
I	20 (16.8 %)

Complication	N (%)	CCS	Treatment
*Early pouch-related complications*			
Major			
Urosepsis	3 (2.5 %)	IVb	Intensive care
Ureteroneovesical stenosis	2 (1.7 %)	IIIb	Re-implantation (1×) Transureteroureterostomy (1×)
Enteroneovesical fistula	1 (0.8 %)	IIIb	Open surgery
Neovesicocutaneus fistula	1 (0.8 %)	IIIb	Endoscopic examination
Neobladder bleeding	1 (0.8 %)	IIIb	Endoscopic examination + Rinsing
Mucus retention	1 (0.8 %)	IIIa	Endoscopic examination + Rising
Minor			
Urinary tract infection (pyelonephritis, pouchitis, fever after removal of single-J stents)	14 (11.8 %)	II	Antibiotics
Clot retention	1 (0.8 %)	II	Rinsing + Antibiotics
Mucus retention	2 (1.7 %)	I	Rinsing
Urinoma	1 (0.8 %)	I	Prolonged drainage
*Early non-pouch-related complications*			
Major			
Death (small bowel perforation resulting in septic shock and myocardial infarction)	1 (0.8 %)	V	Open surgery (laparotomy) + Intensive care
Mesenterial bleeding	1 (0.8 %)	IIIb	Open surgery
Evisceration	1 (0.8 %)	IIIb	Open surgery
Textilloma	1 (0.8 %)	IIIb	Open surgery
Peptic ulcer	1 (0.8 %)	IIIa	Gastroscopy
Pneumothorax	1 (0.8 %)	IIIa	Chest drain
Minor			
Pneumonia	5 (4.2 %)	II	Antibiotics
Catheter sepsis	4 (3.4 %)	II	Antibiotics
Lung embolism	3 (2.5 %)	II	Low molecular weight heparin
Ileus	2 (1.7 %)	II	Total parenteral nutrition (1×) Jejunal catheter (1×)
Fever (unknown origin)	1 (0.8 %)	II	Antibiotics
Back pain	1 (0.8 %)	II	Epidural infiltration
Acidosis	1 (0.8 %)	II	Intravenous bicarbonate
Deep venous thrombosis	1 (0.8 %)	II	Low molecular weight heparin
Wound abscess	1 (0.8 %)	II	Drainage + Antibiotics
Wound problems (dehiscence, fat necrosis)	5 (4.2 %)	I	Vacuum therapy
Ileus	5 (4.2 %)	I	Conservative
Delirium	3 (2.5 %)	I	Oral medication
Diarrhea	3 (2.5 %)	I	Conservative
Catheter sepsis	1 (0.8 %)	I	Removal of deep venous catheter

#### Late complications

Table 3 gives an overview of all late complications and their management. We registered 56 major complications (CCS III–V) in 111 patients and 39 minor complications (CCS I–II). Late pouch-related complications were detected in 47 of 111 patients (42.3 %), most of which were infectious or obstructive (neovesicourethral or ureteroneovesical). Urosepsis was diagnosed in 3 patients (2.7 %) and relapsing UTIs or pyelonephritis in 26 (23.4 %). Neovesicourethral stenosis (NUS) was noted in 9.9 % (n = 11), acute urinary retention due to mucus clot in 5.4 % (n = 6), and ureteroneovesical stenosis in 5.4 % (n = 6). In two patients, a renal unit was lost because of long-term ureteroneovesical obstruction.

**Table 3:** Overview of late complications (>3 months) (pouch and non-pouch-related) by the Clavien–Dindo classification in 111 patients

Clavien–Dindo score (CCS)	Number of complications (%)
Major	
V	1 (0.9 %)
IV	
a	4 (3.6 %)
b	0 (0 %)
III	
a	2 (1.8 %)
b	49 (44.1 %)
Minor	
II	28 (25.2 %)
I	11 (9.9 %)

Complication	N (%)	CCS	Treatment
*Late pouch-related complications*			
Major			
Death (abscess formation in base of penis after false-passage catheterization, resulting in sepsis + multiple organ failure)	1 (0.9 %)	V	Debridement + Intensive care
Loss of renal unit	2 (1.8 %)	IVa	Conservative
Ischemic perforation of neobladder	1 (0.9 %)	IVa	Laparotomy + Intensive care
Urosepsis	1 (0.9 %)	IVa	Intensive care + Conservative therapy
Pouch calculi	8 (7.2 %)	IIIb	Laser lithotripsy
Neovesicourethral stenosis	11 (9.9 %)	IIIb	Internal optical urethrotomy (4×) Transurethral resection (5×) Dilatation under general anesthesia (2×)
Ureteroneovesical stenosis	6 (5.4 %)	IIIb	Ureteral reimplantation (4×) Lifelong single-J stenting (2×)
Stress incontinence	2 (1.8 %)	IIIb	Single incision mid-urethral sling (1×) Fascia lata sling (1×)
Mucus obstruction	1 (0.9 %)	IIIb	Endoscopic evaluation
Neovesicocutaneous fistula	1 (0.9 %)	IIIb	Open excision
Neovesicorectal fistula	1 (0.9 %)	IIIb	Laparotomy + Conversion to ileal conduit
Hypercontractile neobladder with severe incontinence	1 (0.9 %)	IIIb	Laparotomy + Conversion to ileal conduit
Ureterolithiasis	1 (0.9 %)	IIIa	Nephrostomy
False-passage catheterization	1 (0.9 %)	IIIa	Endoscopic evaluation
Minor			
Relapsing urinary tract infections and pyelonephritis	26 (23.4 %)	II	Antibiotics
Urosepsis	2 (1.8 %)	II	Antibiotics + Fluid resuscitation
Mucus obstruction	5 (4.5 %)	I	Conservative
*Late non-pouch-related complications*			
Major			
Wound herniation	17 (15.3 %)	IIIb	Open repair
Small bowel obstruction	1 (0.9 %)	IIIb	Open adhesiolysis
Minor			
Diarrhea	6 (5.4 %)	I	Conservative

Late non-pouch-related complications were registered in 22 patients (19.8 %). Herniation of the laparotomy scar was surgically corrected with a mesh in 17 patients.

### Pouch survival

Four patients had their N-pouch removed. Urethral tumor recurrence was noted in two patients in whom salvage urethropouchectomy and construction of an IC was performed. The other indications were neobladder hyper-contractility and neobladder–rectal fistula in a patient who underwent previous radiotherapy for prostate cancer.

Urethral tumor recurrence was also diagnosed in another two patients, who had endoscopic resection of the lesions. All four patients with urethral tumor recurrence remained alive until last follow-up.

### Continence rates

**Fig. 2:**
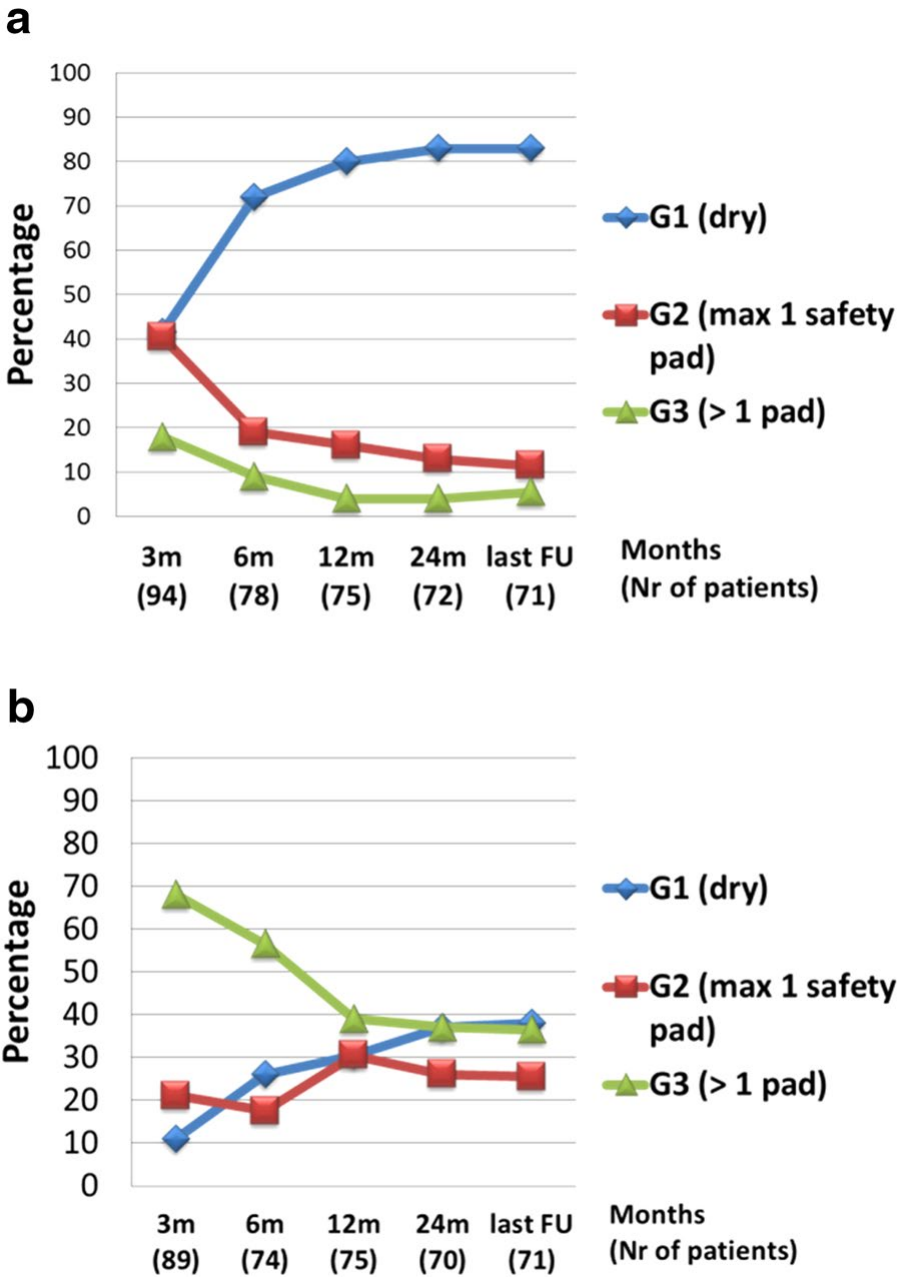
Male continence. **a** Daytime male continence. **b** Nighttime male continence

Among the male patients, for DTC at the 3-month interval, 41.5 % of 94 patients were in G1, 40.5 % in G2, and 18 % in G3. At last follow-up, these rates were 83.1, 11.3, and 5.6 %, respectively, in 71 patients (Fig. 2a). For NTC at the 3-month interval, 11.2, 21.3, and 67.5 % of 89 male patients were in G1, G2, and G3, respectively. Of 71 patients evaluated at last follow-up, these values were respectively 38, 25.4, and 36.6 %. Of 26 incontinent patients (G3), 20 used diapers or condom catheters (Fig. 2b).

**Fig. 3:**
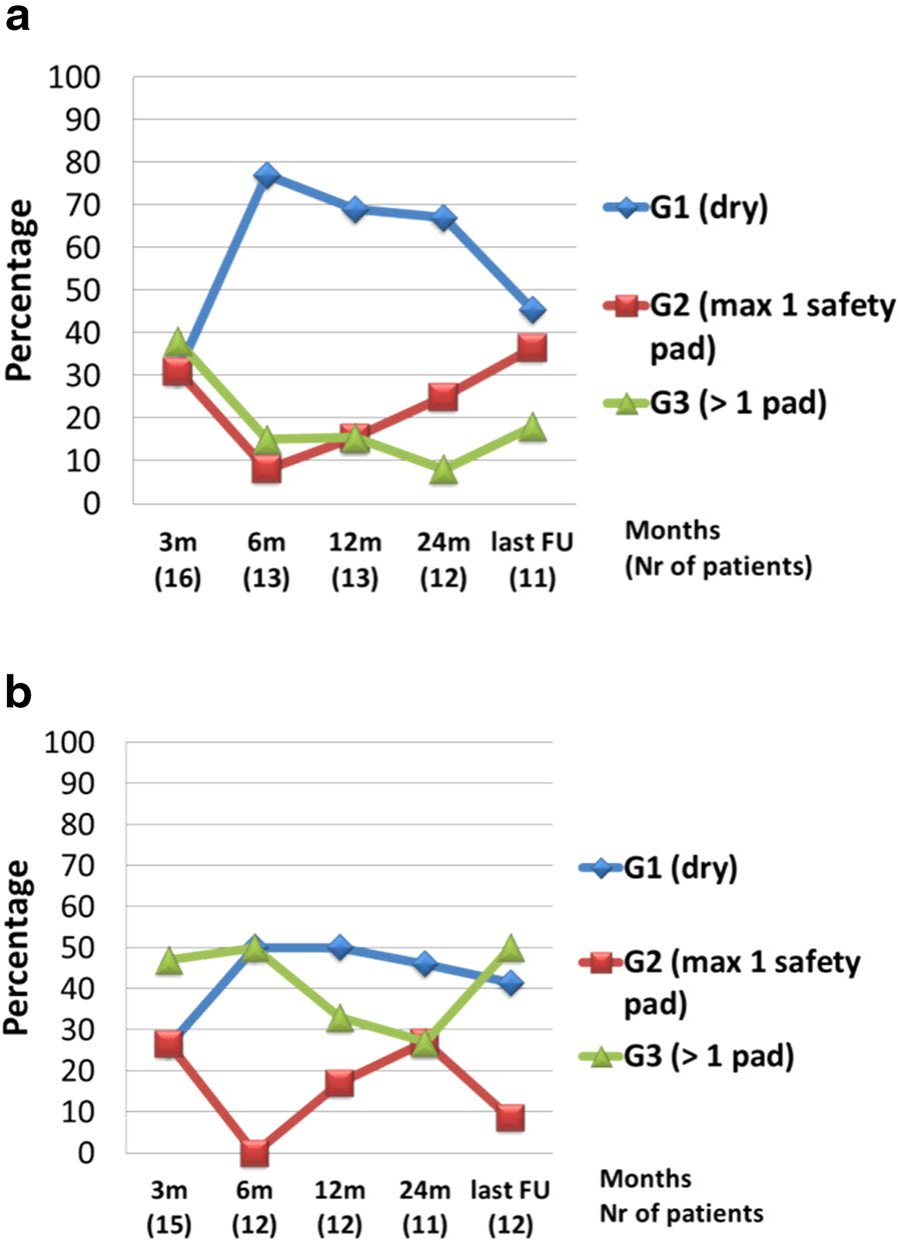
Female continence. **a** Daytime female continence. **b** Nighttime female continence

Among the women, for DTC at the 3-month interval, 31.25, 31.25, and 37.5 % of 16 patients were in G1, G2, and G3, respectively; these percentages were 45.5, 36.3, and 18.2 % of 11 patients at last follow-up. Of note, the two incontinent patients at last follow-up eventually underwent a sling procedure for deteriorating stress incontinence (Fig. 3a). Regarding NTC at the 3-month interval, 26.6, 26.6, and 46.8 % of 15 women were respectively in G1, G2, and G3, whereas these rates were 41.6, 8.4, and 50 %, respectively, for 12 patients at last follow-up. Five out of six women in G3 used diapers (Fig. 3b).

### Hypercontinence and catheterization

Clean intermittent self-catheterization was necessary in 8 out of 17 female patients (47 %), and none of them could void spontaneously. With the exclusion of six patients because of death or loss to follow-up, 18 out of 99 (18 %) male patients required catheterization of whom 12 could still void spontaneously. Of these hypercontinent men, 5 (28 %) had NUS, 2 (11 %) had mucus retention while 11 (61 %) had no clear reason. In the female hypercontinent population, no NUS was observed.

## Discussion

Urinary diversion after RC can be performed in various ways. For OIN, numerous techniques with different intestinal segments and configurations have been described. The 2014 update of the European Association of Urology guidelines in muscle-invasive and metastatic bladder cancer recommends performing an OBS for both male and female patients who do not have any contraindication or tumor in the urethra (grade B recommendation) (Witjes et al. 2014). Nevertheless, population-based studies from Sweden and the United States indicate that OBS is being performed much less frequently than IC, with a rate of approximately 15 % (Jahnson et al. 2008; Gore et al. 2009). OBS is generally preserved for highly motivated and cognitively capable patients. In a 17-year period, we performed 119 (14.7 % of total) consecutive cystectomies with OBS; in the same time span, 691 patients underwent IC in our institution. These relative numbers emphasize our thorough healthier and younger patient selection.

Cystectomy with OBS is an extensive surgery, and complications are therefore common. The current results indicate early complications in more than one-third and late complications in more than half of patients. Especially, the late complication rates seem worse compared to other large series. Early complication rates ranging from 12.6 to 33.6 % and late complication rates between 23.8 and 32 % have been reported at long-term follow-up (Studer et al. 2006; Hautmann et al. 1999; Shaaban et al. 2003; Stein et al. 2004; Meyer et al. 2009). However, high rates of 44 and 61 % for early and 48 and 51 % for late complications have also been reported (Jensen et al. 2006; Nieuwenhuijzen et al. 2008). The relatively long average follow-up time of 78.7 months may partially explain our elevated late complication rate. Almost all patients return for follow-up visits at our center where a very explicit and detailed history is noted in regular follow-up visits. This approach may have led to an increased detection of (minor) complications.

A more nuanced view emerges when we study these complications in detail. Of the 69 early complications encountered in 119 patients, only 15 (21.7 %) were major according to the CCS; thus, major early complications seem to be rather sporadic. Of the 95 late complications in 111 patients, 56 (58.9 %) were considered major (CCS III–V). In contrast to early complications, a significant portion of the late complications seemed to be serious. For example, 15.3 % of patients had open surgical repair of a wound herniation (1.5–6.4 % in other series) (Studer et al. 2006; Hautmann et al. 1999, 2011). Our NUS rate was 9.9 % whereas other large series have yielded stenosis rates ranging from 2.2 to 3.7 % (Studer et al. 2006; Hautmann et al. 1999). This rate is noteworthy because generally a simple buttonhole anastomosis was created, so that urethral stenosis rates would be expected to be similar to other series. These patients underwent re-intervention under (general) anesthesia (urethrotomy, transurethral resection and dilatation).

The late ureteroneovesical stenosis rate in this series was 5.4 %. Most patients underwent ureteral reimplantation, and only two had a lifelong single-J stenting. The ureteroneovesical anastomosis was created by a simple refluxing end-to-end anastomosis, which is believed to have the lowest stenosis rates (Roth et al. 1997). An anti-reflux anastomosis is not considered necessary for three reasons: An OIN is a low-pressure system that cannot provoke active reflux; the afferent ileal limb acts as an anti-reflux protection mechanism; and urine in an OIN is considered to be sterile (Hautmann et al. 2006). Stenosis rates are higher in anti-reflux valve anastomoses (9 %) while refluxing anastomoses stenosis rates are generally reported in no more than 3 % (Studer et al. 2006; Hautmann et al. 1999; Gakis and Stenzl 2010). Long-term and adequate follow-up could again explain the higher stenosis rate in our series.

Of particular interest are the continence rates. DTC rates are generally excellent in large series; continence is mostly achieved in more than 90 % of patients (both male and female), although definitions of continence may differ (Studer et al. 2006; Hautmann et al. 1999; Litwin et al. 1998; Shaaban et al. 2003; Abol-Enein and Ghoneim 2001). When pragmatically considering G1 and G2 (maximum one pad a day) as being continent, DTC reaches up to 96 % in this series, consisting predominantly of men. At the 3-month interval, 82 % of male patients were continent, and this rate gradually reached a plateau at 95 %.

The female population in this study was small; therefore, no strong conclusions can be drawn for this patient group. During the first 6 months, DTC rates matched those of the male population. There seemed, however, to be a more substantial portion of female patients who continued to need a (safety) pad (G2), a number that continued to rise with longer follow-up. The steep rise in incontinence rates (G3) towards the last follow-up can probably be explained by de novo stress incontinence. Two female patients had sling surgery (many years after cystectomy) due to deteriorating stress incontinence.

At the 3-month interval, only 32 % of male and 53 % of female patients achieved NTC. Generally NTC improves more slowly than DTC (Hautmann et al. 1999). NTC rates in male and female patients (including G1 and G2) were 63 and 73 %, respectively, at the 2-year interval, thereafter reaching a plateau.

We present good DTC rates (Figs. 2a, 3a) for the male patients, which are comparable to other series (Studer et al. 2006; Hautmann et al. 1999; Shaaban et al. 2003; Gakis and Stenzl 2010; Abol-Enein and Ghoneim 2001). Our NTC rate of approximately 60–70 % (Figs. 2b, 3b) appears to be lower than the other series (Studer et al. 2006; Abol-Enein and Ghoneim 2001), but a formal comparison is not possible because the definition of incontinence is pragmatic and subjective, and methods and follow-up times for assessing incontinence vary. In particular, nighttime incontinence has no clear definition in most series and can be influenced by nocturnal voiding, patient motivation and neobladder capacity. Perhaps these factors explain the rather low but probably realistic NTC rates in this report. Unfortunately, we have no accurate data on nightly micturition rates, as frequent nightly micturition could improve nighttime incontinence rates.

As some authors have noted, hypercontinence is not an infrequent problem, especially among female patients, with rates of 16–33 % (Arai et al. 1999; Lee et al. 2004). This high percentage particularly in women can be due to kinking of the neobladder at the level of bladder neck due to a wider pelvis, absence of posterior support by the uterus, or injury of the autonomous nerves of the urethra. Most hypercontinent men remain able to void spontaneously, while women do not. All patients should be willing to self-catheterize before undergoing surgery. Because of the risk for nightly incontinence and self-catheterization, OBS should be recommended only to highly motivated patients.

This study has some limitations. With the retrospective design, we could not evaluate all patients for all data because of some deaths and missing data. Moreover, we acknowledge complication underreporting in retrospective studies, and complications treated outside our center could have been missed. We used the CCS to standardize complication reporting and differentiate major and minor complications. Although this classification was not originally developed for complications seen later than 90 days after surgery, CCS appears to be reasonably appropriate for reporting long-term complications; however, Hautmann et al. (2011) concluded that it seemed less suited for doing so. Nevertheless, there is no specific scoring system dedicated solely to the early/late complications of RC and urinary diversion. Our rather high complication rates could arise from very accurate data gathering that, combined with a long follow-up, possibly has yielded more realistic rates than other series. As Donat (2007) proposed and Shabsigh et al. (2009) demonstrated, meticulously reporting short- and long-term complications gives better information about true incidence and outcome not only in RC series but also in other uro-oncological procedures.

## Conclusion

We present a retrospective analysis of 119 patients who underwent the Leuven N-pouch with long-term follow-up. Complication rates were relatively high, reflecting a strict and meticulous follow-up scheme. Good DTC rates for male patients were achieved, but NTC rates were less than ideal. High complication and nighttime incontinence rates strongly support reserving OBS only for thoroughly selected patients who can self-catheterize .

## Abbreviations

RC: radical cystectomy
OIN: orthotopic ileal neobladder
IC: ileal conduit
OBS: orthotopic bladder substitution
CCS: Clavien–Dindo classification score
UCLA: University of California, Los Angeles
G: group
DTC: daytime continence
NTC: nighttime continence
UTI: urinary tract infection
NUS: neovesicourethral stenosis
